# Cannabinoid-deficient Benin republic hemp (*Cannabis sativa* L.) improves semen parameters by reducing prolactin and enhancing anti-oxidant status

**DOI:** 10.1186/s12906-019-2541-5

**Published:** 2019-06-17

**Authors:** Abdullateef Isiaka Alagbonsi, Luqman Aribidesi Olayaki, Halimat Amin Abdulrahim, Thomson Sijuade Adetona, Gbemileke Tobiloba Akinyemi

**Affiliations:** 1 Department of Clinical Biology (Physiology), School of Medicine and Pharmacy, University of Rwanda College Medicine and Health Sciences, Huye, Rwanda; 2 Department of Physiology, College of Health Sciences, University of Ilorin, Kwara, Nigeria; 3 Department of Medical Biochemistry, College of Health Sciences, University of Ilorin, Kwara, Nigeria

**Keywords:** *Cannabis sativa*, Fatty acids, Hyperprolactinemia, Hypothalamic-pituitary-testicular axis, Oxidative stress, Semen parameters

## Abstract

**Background:**

Nigerian *Cannabis sativa* (hemp) causes male gonadotoxicity by inducing hyperprolactinemia, down-regulation of hypothalamic-pituitary-testicular axis, and oxidative stress. Benin republic hemp has been preferred by illicit users in Nigeria but its effect on male fertility is not understood. This study determined and compared the compositions of Benin republic hemp ethanol extract (BHE) and Nigerian hemp. The effects of BHE on semen parameters, reproductive hormones, and anti-oxidant status, and the possibility of bromocriptine (prolactin inhibitor) to abolish hemp-induced toxicities in rats were also investigated.

**Methods:**

Thirty-six male Wistar rats were blindly randomized into 6 oral treatment groups (*n* = 6 each). Groups I (control) and II received normal saline and bromocriptine (3 mg/kg) respectively. Groups III and IV received 2 mg/kg of BHE alone and in combination with bromocriptine respectively, while groups V and VI received 10 mg/kg BHE alone and in combination with bromocriptine respectively. Comparisons among the groups were done by one-way analysis of variance, followed by *post-hoc* Tukey multiple comparison test. Statistical significance was considered at *p* < 0.05.

**Results:**

The BHE has no cannabichromene and tetrahydrocannabinol but a very small quantity of cannabinol and higher quantity of fatty acids when compared to Nigerian hemp. Both doses of BHE increased sperm count, morphology and viability but not motility. Co-administration of BHE with bromocriptine lowered sperm count but increased sperm morphology and viability. Bromocriptine and/or BHE caused reduction in the plasma prolactin level, increase in the plasma superoxide dismutase activity, but no significant change in the plasma gonadotropin releasing hormone, follicle stimulating hormone (except for the increase in rats that received bromocriptine+ 10 mg/kg BHE), luteinizing hormone, estradiol, malondialdehyde and glutathione peroxidase. The 10 mg/kg BHE or bromocriptine+BHE (both doses) increased total anti-oxidant capacity and catalase.

**Conclusions:**

The BHE improves semen parameters by reducing plasma prolactin and enhancing plasma anti-oxidant status. Its pro-fertility potential might be associated with its deficiency in the widely known gonadotoxic phytocannabinoids.

## Background

*Cannabis sativa* (hemp), the most widely abused recreational drug worldwide [1], is becoming globally legalized due to its medicinal use by patients with epilepsy [2], inflammation [3], cancer [4], pain [5], etc. Furthermore, many synthetic compounds that act on the endocannabinoid system have been extensively studied in recent years [6–8]. However, its detrimental effects on the male reproductive functions have been well-established. For instance, it has been reported to decrease semen parameters [9–11], germ cell proliferation and reproductive organ weight [10, 12]. Mechanisms including hyperprolactinemia [13], down-regulation of hypothalamic-pituitary-testicular (HPT) axis [13, 14], endocrine disruption, and oxidative stress [8] have been implicated in hemp-induced gonadotoxicity.

Hyperprolactinemia has been reported to have a definite role in male infertility. It causes infertility in about 11% of oligospermic males [15] by inhibiting gonadotropin releasing hormone (GnRH) pulsatile secretion**,** which impliedly decreases gonadotropins (luteinizing hormone [LH] and follicle stimulating hormone [FSH]) and testosterone secretion. This prolactin-induced down-regulation of HPT axis ultimately leads to spermatogenic arrest, altered sperm quality, impaired sperm motility, and later produces secondary hypogonadism and male infertility. In addition to this indirect effect, hyperprolactinemia also directly influences steroidogenesis and spermatogenesis by acting on prolactin receptors present in Leydig and Sertoli cells in the testes to produce primary hypogonadism and male infertility [15].

There have been evidences that the hyperprolactinemia-induced infertility is reversible [15–18]. A simple medication like bromocriptine or cabergoline, which normalizes serum prolactin levels, has been consistently shown to restore gonadal functions, reverse infertility caused by hyperprolactinemia, and reduces prolactinoma size in majority of patients [17–19]. Thus, we also speculated that the hemp-induced gonadotoxicity mediated through hyperprolactinemia and down-regulation of HPT axis [13] might be reversed or prevented by inhibiting endogenous prolactin secretion with bromocriptine.

The National Drug Law Enforcement Agency (NDLEA) of Nigeria, through series of online and print media publications, has recently observed that most of the illegal hemp users in Nigeria have been preferentially obtaining hemp from Benin republic. The NDLEA also observed that users now compress this imported hemp and soak it with ethanol prior to its use; and that the “high” and euphoria felt by users of Nigerian and Benin republic hemp are comparatively different. It is not yet known whether there is difference in the quality of both hemp samples or not. The present study determined and compared the compositions of Benin republic hemp ethanol extract (BHE) and Nigerian hemps.

The effects of BHE on semen parameters, reproductive hormones, and anti-oxidant status were also investigated in rats. Bearing the prolactin-mediated gonadotoxic effect of Nigerian hemp [13] in mind, we further investigated if inhibition of prolactin with bromocriptine could abolish hemp-induced toxicities.

## Methods

### Animals

Thirty-six (36) adult male Wistar rats (weight range: 160–180 g) were obtained from the Department of Biochemistry, University of Ilorin, Nigeria. They were housed in wooden cages maintained under standardized conditions (12-h light/dark cycle, 27–30 °C, 50–80% relative humidity), and were acclimatized in the laboratory for 2 weeks before the commencement of the study. The rats were fed with standard pelletized rodent diet (Ace Feeds, Ibadan, Nigeria) and water ad libitum. All the animals were well-catered for according to the criteria outlined in the ‘Guide for the Care and Use of Laboratory Animals’ prepared by the National Academy of Science and approved by the Ethical Research Committee of the University of Ilorin, Nigeria.

### Extraction and GC-MS analyses of hemp samples

Mixture of hemp seeds and leaves (75%:15% respectively), both of Benin republic and Nigeria origin, were kindly donated by the National Drug Law Enforcement Agency (NDLEA), Nigeria, for research purpose only. About 200 g of each of these samples was subjected to extraction with 98% ethanol in Soxhlet apparatus for 4–8 h as described earlier [20]. The extract was evaporated to dryness in a rotary evaporator under vacuum to get the oil.

A gas chromatography (GC) from AgilentCo. USA, hyphenated to a mass spectrometer (MS, 5975C) with triple axis detector equipped with an auto-injector (10 μl syringe) was used. Helium gas was used as a carrier gas. All chromatographic separation was performed on capillary column having the following specifications: length- 30 m, internal diameter- 0.25 μm, thickness- 250 μm, treated with phenylmethylsilox. Other gas chromatography mass spectrometry (GC-MS) conditions are: ion source temperature (EI)- 250 °C, interface temperature- 300 °C, pressure- 16.2 psi, cut time- 1.8 min, 1.0 μL injector in splitless mode with split ratio 1:50 and injection temperature of 300 °C. The column temperature started at 35 °C for 5 min and changed to 150 °C at the rate of 4 °C/min for 2 min. The temperature was raised to 250 °C at the rate of 20 °C/min and held for 5 min.

The MS scanning was performed from M/Z85 to M/Z380. The GC-MS solution software provided by supplier was used to control the system and to acquire the data. The separation of the injected samples was carried out on an HP capillary column (HP-5MS). Identification of the compounds was carried out by comparing the mass spectra obtained with those of the standard mass spectra from National Institute for Standards and Technology (NIST) library (NIST II). Identification was based on the molecular structure, molecular mass and calculated fragments. The fatty acids and other organic compounds were identified by comparing their retention times with those of the standards. The quantity of each compound present was expressed as percentage of the total compounds.

### Experimental design

Since the hemp sample of Nigerian origin has been repeatedly used in the series of our previous studies [10, 11, 13], we did not repeat its administration in this study, but only compared its composition with that of Benin republic. The 36 rats were blindly randomized into 6 groups (*n* = 6 per group). Groups I (control) and II received normal saline and bromocriptine (3 mg/kg) respectively. Groups III and IV received 2 mg/kg of BHE [10, 11] alone and in combination with bromocriptine [21] respectively, while groups V and VI received 10 mg/kg BHE [22] alone and in combination with bromocriptine respectively. All treatments were given once daily for a period of 30 days between 8:00 am to 10:00 am via oral gavage.

Animals were anaesthetized a day after the last treatment with sodium pentobarbital (40 mg/kg body weight, *im*). Thereafter, they were dissected in order to collect blood by cardiac puncture. Whole blood for the determination of the biochemical markers were collected in heparinized sample bottles which were centrifuged at 3500 rpm for about 10 min at -4 °C using a cold centrifuge (Model 8881, manufacturer: Centurion Scientific Ltd., United Kingdom). The separated plasma samples were collected into separate plain bottles and were stored at -20 °C prior to the biochemical analyses.

### Determination of semen parameters

The procedure for determination of epididymal sperm parameters, such as; count, motility, morphology, and viability are summarized below as previously described [23, 24],

The testes from each rat were carefully exposed and one of them was removed together with its epididymis. For each separated epididymis, the caudal part was removed and placed in a beaker containing 1 ml of normal saline solution. It was macerated with a pair of sharp scissors and left for few minutes to liberate the sperm cells into the normal saline. Semen drops were placed on a clean grease-free glass slide and two drops of warm 2.9% sodium citrate were added. The improved Neubauer counting chamber was charged with the semen solution and the number of sperm cells, appearing as black dots, was counted in 25 small squares within the central counting area of the counting chamber.

The sperm suspension was diluted in 1 ml of normal saline solution. About 10 L was pipetted onto a clean grease free glass slide. A cover slip was lowered onto the sample on the slide, avoiding air bubbles, and the slide was examined using a microscope with a 40X objective. At least, six widely spaced fields were examined to provide an estimate of the percentage of the progressively motile sperm cells. The sperm cells with progressive motility were estimated and recorded as (N) while the total number of all the sperm cells counted was recorded as (T). Sperm motility (%) was calculated using (N/T × 100%).

The principle for determination of sperm morphology was based on the ability of morphologically normal sperm to appear white in color as the plasma membrane will prevent the dye to enter, while abnormal sperms take up the dye and stain dark color. The microscope slides and the eosin stain were pre-warmed to room temperature. One milliliter of the sperm suspension – normal saline solution was transferred to a test tube and 2 drops of 1% eosin were added and mixed gently for agitation. This was incubated for 45–60 min to allow its proper staining and then re-suspended with a Pasteur pipette. A clean grease-free glass slide was used. Potential damage to the sperm cells should be avoided. One or 2 drops of the stained sperm were placed approximately 1 cm from the end of the slide lying on a flat surface. A second slide was held with the slide’s long edge gently touching across the width of the sperm slide and pulled across to produce a sperm smear. After air-drying the slide, using a microscope at 100X objective, the sperm cells were examined. The sperm along the periphery was normally excluded from the examination because there is a greater tendency for artifacts to occur in these regions. At least, five fields were viewed covering the whole slide. Examples of morphological abnormalities are double-headed, elongated head, pyriform head, bent head, bent tail, bent mid-piece, coiled tail, double tail, headless, tailless, etc. All those with normal morphology were recorded as N while the total number of the counted spermatozoa was recorded as T. The percentage sperm morphology was calculated as (N/T × 100%).

### Determination of biochemical parameters

Diagnostic kits for the determination of malondialdehyde (MDA), total anti-oxidant capacity (TAC), superoxide dismutase (SOD), glutathione Peroxidase (GPx), and catalase were obtained from Fortress Diagnostics Limited, United Kingdom. In addition, analytic kits for the determination of GnRH, prolactin, FSH, LH, testosterone, and estradiol were procured from Elabscience Biotechnology Company Ltd. Wuhan, Hubei, China. The analyses were done with spectrophotometer (Spectramax Plus; Molecular Devices, Sunnyvale, CA, USA) according to the manufacturers’ instructions.

### Data analyses

Statistical analyses were done using statistical package for social sciences (SPSS) version 16.0 (IBM Corporation, Armonk, NY). Values were expressed as Mean ± S.E.M. of the variables measured. Comparisons among the groups were done by one-way analysis of variance (ANOVA), followed by *post-hoc* Tukey multiple comparison test. Statistical significance was considered at *p* < 0.05

## Results

### Organic compositions of hemp samples identified by GC-MS

The BHE is rich in stearic acid (27.98%), followed by benzoic acid (14.43%), oleic acid (11.51%), benzeneacetic acid (10.72%), and benzophenone azine (9.96%). It has intermediate quantity of aniline (3.18%), cyclohexanecarboxylic acid (4.75%), and stearic glycidol (3.81%) but lower quantity of 2,3,4- Trimethoxyphenylethylamine (0.35%), Benzamide (0.29%), palmitic acid (1.09%), and cannabinol (1.20%). The acetone fraction from the ethanol extract of Nigerian hemp has its highest constituent as cannabinol (36.5%), followed by tetrahydrocannabinol (THC, 19.81%), α-linolenic acid (12.13%), palmitic acid (8.91%), oleic acid (5.94%), linoleic acid (4.87%), cannabichromene (1.7%), and stearic acid (1.42%). However, the n-hexane fraction from the ethanol extract of Nigerian hemp has its highest constituent as THC (49.11%), followed by cannabinol (20.25%), cannabichromene (5.58%), and linoleic acid (4.31%) (Table 1).

**Table 1:** Organic composition of hemp samples revealed by GC-MS

S/N	Chemical constituents	% Composition in Benin republic hemp ethanol extract	% composition in Nigerian hemp (acetone fraction)	% composition in Nigerian hemp (n-hexane fraction)
1	Benzoic acid	14.43	–	–
2	Aniline	3.18	–	–
3	2,3,4- Trimethoxyphenylethylamine	0.35	–	–
4	Benzamide	0.29	–	–
5	Palmitic acid	1.09	8.91	–
6	Cyclohexanecarboxylic acid	4.75	–	–
7	Stearic acid	27.98	1.42	–
8	Oleic acid	11.51	5.94	–
9	Benzophenone azine	9.96	–	–
10	Stearic glycidol	3.81	–	–
11	Benzeneacetic acid	10.72	–	–
12	Alpha-linolenic acid	–	12.13	–
13	Linoleic acid	–	4.87	4.31
14	Cannabichromene	–	1.7	5.58
15	Tetrahydrocannabinol	–	19.81	49.11
16	Cannabinol	1.20	36.5	20.25

### Effects of bromocriptine and/or BHE on semen parameters

**Fig. 1:**
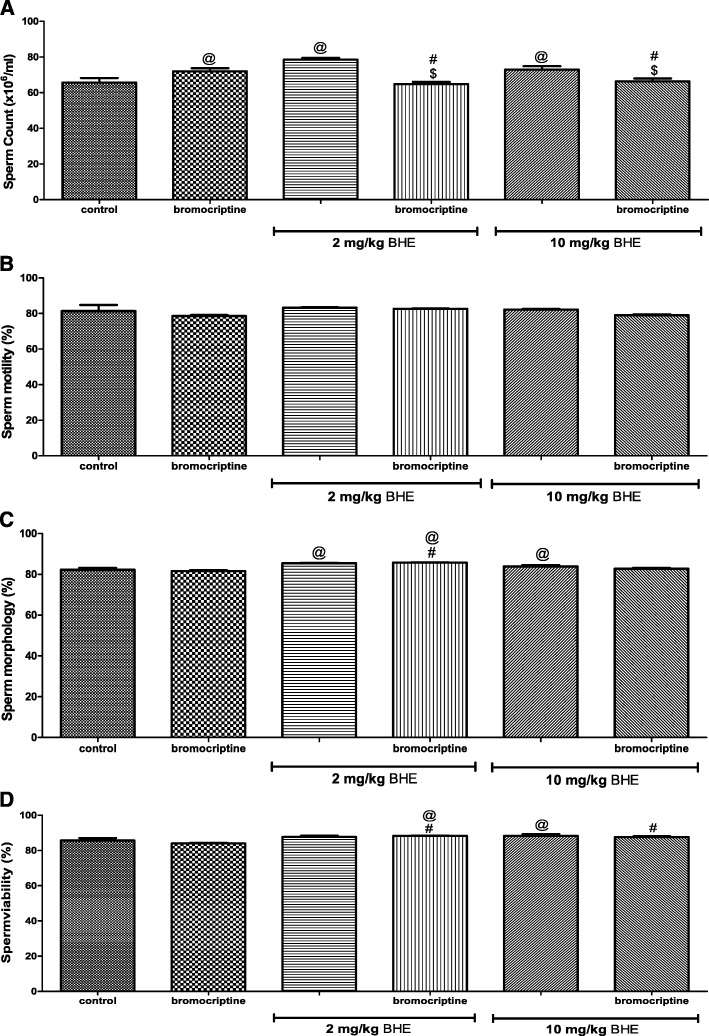
Effects of bromocriptine and/or BHE on semen parameters (**a** Sperm count, **b** sperm motility, **c** sperm morphology, **d**: sperm viability) in rats. BHE denotes Benin republic hemp ethanol extract; ^@^*p* < 0.05 vs. control; ^#^*p* < 0.05 vs. bromocriptine; ^$^*p* < 0.05 vs. corresponding BHE of same dose

Bromocriptine-treated rats had higher sperm count but unchanged sperm motility, morphology and viability when compared to control. Both doses of BHE increased the sperm count, morphology and viability but not sperm motility when compared to control. Rats that were co-administered with either dose of BHE and bromocriptine had lower sperm count but higher sperm morphology and viability when compared to rats that received bromocriptine alone (Fig. 1a-d).

### Effects of bromocriptine and/or BHE on reproductive hormones

Treatment of rats with bromocriptine and/or BHE caused reduction in the plasma prolactin level but no significant change in the plasma GnRH, FSH, LH and estradiol (except for the significant increase in FSH of rats that were co-administered with bromocriptine and 10 mg/kg BHE) when compared to control. Neither bromocriptine nor any of the doses of BHE affected the plasma testosterone, but their co-administration significantly increased the plasma testosterone level (Table 2).

**Table 2:** Effects of bromocriptine and/or BHE on reproductive hormones in male rats

	Control	Bromocriptine	2 mg/kg Hemp extract	2 mg/kg Hemp extract + bromocriptine	10 mg/kg Hemp extract	10 mg/kg Hemp extract + bromocriptine
GnRH (pg/ml)	1382.7 ± 45.37	1389.4 ± 23.84	1243.2 ± 168.47	1399.1 ± 29.24	1371.7 ± 87.69	1405.5 ± 11.79
Prolactin (ng/ml)	8.17 ± 1.21	5.26 ± 0.20^@^	6.56 ± 0.37^@^	6.33 ± 0.56^@^	6.18 ± 0.35^@^	5.75 ± 0.47^@^
FSH (mIU/ml)	2.00 ± 0.46	2.25 ± 0.50	2.66 ± 0.53	2.36 ± 0.62	1.75 ± 0.23	6.43 ± 1.76^@#$^
LH (mIU/ml)	0.92 ± 0.05	0.93 ± 0.03	0.92 ± 0.06	0.76 ± 0.04	0.75 ± 0.06	0.80 ± 0.08
Testosterone (ng/ml)	0.47 ± 0.03	0.46 ± 0.03	0.49 ± 0.01	0.61 ± 0.03^@#$^	0.55 ± 0.04	0.65 ± 0.05^@#$^
Oestradiol (pg/ml)	1.96 ± 0.03	1.99 ± 0.08	1.92 ± 0.02	2.20 ± 0.24	1.92 ± 0.05	2.08 ± 0.11

BHE denotes Benin republic hemp ethanol extract; GnRH denotes Gonadotropin releasing hormone; FSH denotes follicle stimulating hormone; LH denotes luteinising hormone

^@^*p* < 0.05 vs. control; ^#^*p* < 0.05 vs. bromocriptine; ^$^*p* < 0.05 vs. corresponding BHE of same dose

### Effects of bromocriptine and/or BHE on lipid peroxidation and anti-oxidant status

**Fig. 2:**
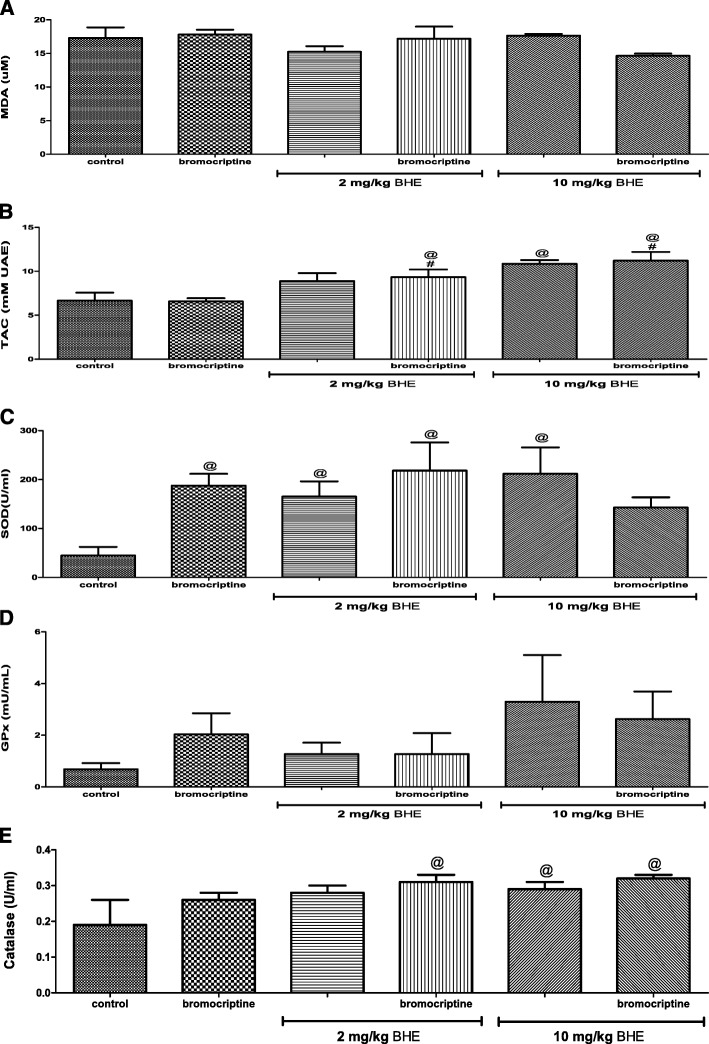
Effects of bromocriptine and/or BHE on lipid peroxidation (**a** malondialdehyde) and anti-oxidant status (**b** total anti-oxidant capacity [TAC], **c** superoxide dismutase [SOD], **d** glutathione peroxidase [GPx], and **e** catalase) in male rats. BHE denotes Benin republic hemp ethanol extract; ^@^*p* < 0.05 vs. control; ^#^*p* < 0.05 vs. bromocriptine; ^$^*p* < 0.05 vs. corresponding BHE of same dose

Treatment of rats with bromocriptine and/or BHE caused increase in the plasma SOD activity but no significant change in the plasma MDA and GPx when compared to control. Rats that received 10 mg/kg of BHE and those that received combination bromocriptine and either dose of BHE had higher TAC and catalase when compared to control (Fig. 2a-e).

## Discussion

Hemp seeds have been developed into numerous products for food, cosmetic, therapeutic, functional food and nutraceutical industries [25]. The presence of polyunsaturated fatty acids (PUFAs) in hemp seed oil has been extensively documented. It contains α-linolenic acid (omega-3 PUFA) and linoleic acid (omega-6 PUFA), both of which are essential fatty acids required to prevent cardio-metabolic disease [26]. It also contains γ-linolenic acid, which makes it as an ideal ingredient for cosmetics (lipid-enriched creams, light body oils, etc.) [27]. Though the present study did not identify PUFAs (α-linolenic and linoleic acids) in BHE as reported by authors that analyzed hemp from other sources [25], we however observed that it is a rich source of saturated (palmitic and stearic acids) and monounsaturated (oleic acid) fatty acids [25].

Because about 35% of the available oil has been reported to remain in the seed cake following pressing process while supercritical carbon dioxide extraction has been documented as a better alternative to pressing or organic solvent methods for extraction of lipids such as PUFAs from fish lipids and vegetable oils [28, 29], the discrepancy in the identified constituents might be thought to result from different methods of extraction: we used solvent extraction while Da Porto et al. [25] used supercritical carbon dioxide. However, this possibility is annulled by their previous observation that there was no difference between the fatty acid composition obtained from hempseed oil following Soxhlet apparatus or by supercritical carbon dioxide method of extraction [25].

We have consistently shown that ethanol extract of hemp leaves of Nigeria origin causes gonadotoxicity in male rats by eliciting hyperprolactinemia, down-regulation of HPT axis, oxidative stress, and endocrine disruption [10, 11, 13]. The improvement in semen parameters by BHE in this study is contrary to our previous reports and expectation, and thus of great surprise and interest to us. Because environmental variables of the soil at different location have been known to affect plant species composition and richness [30], we speculate that the discrepancy in our previous and current findings could result from different origins of the hemp samples used in both studies. We queried this speculation further by determining the GC-MS composition of acetone and n-hexane fractions of the ethanol extract of Nigerian hemp used for our previous studies. We confirmed the presence of α-linolenic acid in the acetone fraction only, but linoleic acid in both fractions. Furthermore, both fractions have cannabinoids including cannabichromene, THC, and cannabinol. It is interestingly worthy of note that these cannabinoids accounts for about 58% of the total composition of the acetone fraction but 75% of the n-hexane fraction of the Nigerian hemp. However, the BHE contains only cannabinol at very negligible quantity (1.2%) but no cannabichromene and tetrahydrocannabinol. Because both hemp samples from these two different locations gave varying GC-MS composition despite the same extraction method (using Soxhlet apparatus), it could be opined that the discrepancy in the composition is not related to the methods of extraction but related to the climatic difference of the origin of both hemp samples.

The beneficial effect of lipids on semen parameters has been well-documented. Cholesterol has been reported to increase cell membrane stability and decrease the transition temperature from the fluid to the gelatinous phase [31]. Phospholipids also contribute to membrane permeability and flexibility, and cell functionality. Because the cholesterol to phospholipids proportion cannot be altered in sperm due to its dependence on species’ genetic factor [32], addition of PUFAs to diets has been suggested in order to increase its proportion relative to sperm membrane phospholipids and improve its fluidity and resistance to thermal stress [33]. Moreover, dietary fatty acids supplementation has been reported to improve the cryopreserved stallion semen [34]. We have also shown in the very recent publication from our laboratory that fatty acids could be responsible for the spermatoprotective effect of *Telfairia occidentalis* leaves in rats [35]. On the contrary, THC (the psychoactive cannabinoid in hemp) has been consistently shown to inhibit motility, capacitation and acrosome reaction of human and boar spermatozoa through cannabinoid receptors 1 and 2 [12, 36–38]. We have also recently observed that THC inhibits the hyper-activated motility of capacitated rat sperms by reducing sperm velocities (average path, curvilinear, and straight-line), amplitude of lateral head displacement, and beat cross frequency [7]. The improvement in semen parameters by BHE could thus be partly associated with its fatty acid constituents and its comparative deficiency in cannabinoids, while the abundant proportion of cannabinoids in Nigerian hemp could have superimposed its fatty acids and lead to gonadotoxicity as previously reported by us [10, 11, 13].

The release of prolactin from adenohypophysis is under tonic inhibition of dopamine, which is delivered to adenohypophysis from the hypothalamus via the hypothalamic-hypophyseal portal system. Dopamine decreases prolactin secretion by binding to the dopamine D2 receptors on the surface of lactotroph and diminishing intracellular cyclic adenosine monophosphate (cAMP) level. We have previously shown that increase in the plasma prolactin level is one of the mechanisms through which Nigerian hemp induces male gonadotoxicity [13]. Therefore, it was tempting to investigate the role of prolactin in the improvement of semen parameters elicited by Benin republic hemp. Bromocriptine is a dopamine agonist that has been used for the treatment of hyperprolactinemia. Administration of bromocriptine alone increased sperm count but did not significantly affect other semen parameters. However, it synergizes with hemp extract to enhance sperm morphology and viability (though there were decreases in sperm count), which tends to involve the FSH and testosterone. Moreover, the plasma level of prolactin was decreased by both doses of the hemp extract and/or bromocriptine, while there were no significant changes in the plasma GnRH, gonadotropins (FSH and LH), and testosterone in hemp-treated rats. Similarly, previous studies have reported normal serum levels of gonadotropins but elevated serum prolactin in azoospermic or oligospermic patients, providing evidence for the sole role of prolactin on spermatogenesis independent of gonadotropins [15, 16]. Our current data also suggest that the pro-fertility potential of Benin republic hemp is partly mediated through inhibition of endogenous prolactin but independent of HPT axis. This is different from the Nigerian hemp that induces gonadotoxicity through hyperprolactinemia and down regulation of HPT axis [13].

Apart from hyperprolactinemia and down-regulation of HPT axis, we have further previously shown that the gonadotoxic effect of Nigerian hemp is also mediated through oxidative stress [10, 11]. In the current study, we investigated if the pro-fertility effect of Benin republic hemp will be related to an enhanced plasma anti-oxidant status. We observed that the hemp extract enhanced the TAC, which is accounted for by SOD and catalase but not GPx. This agrees with a previous study that observed that hemp seed oil has free radical scavenging potential even more than olive oil [39]. Moreover, there was no lipid peroxidation in any of the experimental groups. Like the pattern observed for the sperm morphology and viability, there was a synergistic relationship between bromocriptine and hemp extract to enhance the plasma anti-oxidant status.

## Conclusions

In conclusion, this study shows that hemp sample from Benin republic is deficient in cannabinoids. It also shows that this hemp sample improves semen parameters by reducing plasma prolactin and enhancing plasma anti-oxidant status. It was opined that the pro-fertility effect of the Benin republic hemp sample could be due to its deficiency in the widely known gonadotoxic cannabinoids. This study has cautioned us from making a generalized claim that hemp is gonadotoxic. It shows that the origin of hemp samples might determine their composition and their ethno-pharmacological effects on the body functions.

## Data Availability

The datasets used and/or analyzed during the current study are available from the corresponding author on reasonable request.

## Abbreviations

ANOVA: Analysis of variance
BHE: Benin republic hemp ethanol extract
cAMP: cyclic adenosine monophosphate
FSH: Follicle stimulating hormone
GC-MS: Gas chromatography mass spectrometry
GnRH: Gonadotropin releasing hormone
GPx: Glutathione peroxidase
HPT: Hypothalamic-pituitary-testicular
LH: Luteinizing hormone
MDA: Malondialdehyde
NDLEA: National Drug Law Enforcement Agency
NIST: National Institute for Standards and Technology
PUFA: Polyunsaturated fatty acids
SEM: Standard error of mean
SOD: Superoxide dismutase
SPSS: Statistical package for social sciences
TAC: Total anti-oxidant capacity
THC: Tetrahydrocannabinol

## References

[1] Abdel-Salam O (2016). Gastric acid inhibitory and gastric protective effects of cannabis and cannabinoids. Asian Pac J Trop Med.

[2] Tzadok M, Uliel-Siboni S, Linder I, Kramer U, Epstein O, Menascu S, Nissenkorn A, Yosef OB, Hyman E, Granot D (2016). CBD-enriched medical cannabis for intractable pediatric epilepsy: the current Israeli experience. Seizure.

[3] Juknat A, Kozela E, Kaushansky N, Mechoulam R, Vogel Z (2016). Anti-inflammatory effects of the cannabidiol derivative dimethylheptyl-cannabidiol – studies in BV-2 microglia and encephalitogenic T cells. J Basic Clin Physiol Pharmacol.

[4] Velasco G, Hernandez-Tiedra S, Davila D, Lorente M (2016). The use of cannabinoids as anticancer agents. Prog Neuro-Psychopharmacol Biol Psychiatry.

[5] Lynch ME (2016). Cannabinoids in the management of chronic pain: a front-line clinical perspective. J Basic Clin Physiol Pharmacol.

[6] Lazzari P, Serra V, Marcello S, Pira M, Mastinu A (2017). Metabolic side effects induced by olanzapine treatment are neutralized by CB1 receptor antagonist compounds con-administration in female rats. Eur Neuropsycopharmacol.

[7] Alagbonsi IA, Olayaki LA (2018). Melatonin attenuates ∆^9^-tetrahydrocannabinol-induced reduction in rat sperm motility and kinematics *in-vitro*. Reprod Toxicol.

[8] Bonini SA, Premoli M, Tambaro S, Kumar A, Maccarinelli G, Memo M, Mastinu A (2018). Cannabis sativa: a comprehensive ethnopharmacological review of a medicinal plant with a long history. J Ethnopharmacol.

[9] Gundersen TS, Jorgensen N, Andersson A, Bang AK, Nordkap L, Shakkebak NE, Priskorn L, Juul A, Jensen TK (2015). Association between use of marijuana and male reproductive hormones and semen quality: a study among 1,215 healthy young men. Am J Epidemiol.

[10] Alagbonsi IA, Olayaki LA, Salman TM (2016). Melatonin and vitamin C exacerbate *Cannabis sativa*-induced testicular damage when administered separately but ameliorate it when combined in rats. J Basic Clin Physiol Pharmacol.

[11] Alagbonsi IA, Olayaki LA (2017). Role of oxidative stress in *Cannabis sativa-*associated spermatotoxicity: evidence for ameliorative effect of combined but not separate melatonin and vitamin C. Middle East Fertil Soc J..

[12] Cobellis G, Cacciola G, Scarpa D, Meccariello R, Chianese R, Franzoni MF, Mackie K, Pierantoni R, Fasano S (2006). Endocannabinoid system in frog and rodent testis: type-1 cannabinoid receptor and fatty acid amide hydrolase activity in male germ cells. Biol Reprod.

[13] Alagbonsi IA, Olayaki LA (2016). Ameliorative effect of combined melatonin and vitamin C on *Cannabis sativa-*induced reproductive hormonal toxicity. J Afr Ass Physiol Sci.

[14] El-Habashi AA, Mousa MA, El-Eraky WI, Khalil WKB, Ahmed HH, Moad MAA (2013). Possible mechanisms for the toxic effects of marijuana smoke on the reproductive axis of male albino rats. J Appl Pharm Sci.

[15] Masud S, Mehboob F, Bappi MU (2007). Severe hyperprolactinemia directly depresses the gonadal activity causing infertility. Esculapio J Services Inst Med Sci.

[16] Soler Fernández JM, Caravaca Magariños F, Domínguez Bravo C, Murillo Mirat J, Aparicio Palomino A, Herrera Puerto J (1990). Correlation of serum prolactin, sperm count and motility. Prevalence of hyperprolactinemia in the infertile male. Arch Esp Urol.

[17] Buvat J (2003). Hyperprolactinemia and sexual function in men: a short review. Int J Impot Res.

[18] De Rosa M, Zarrilli S, Di Sarno A, Milano N, Gaccione M, Boggia B, Lombardi G, Colao A (2003). Hyperprolactinemia in men: clinical and biochemical features and response to treatment. Endocrine.

[19] Laufer N, Yaffe H, Margalioth EJ, Livshin J, Ben-David M, Schenker JG (1981). Effect of bromocriptine treatment on male infertility associated with hyperprolactinemia. Arch Androl.

[20] Mandal TK, Das NS (2010). Testicular toxicity in cannabis extract treated mice: association with oxidative stress and role of anti-oxidant systems. Toxicol Ind Health.

[21] Dall’Ara A, Lima L, Cocchi D, Di Salle E, Cancio E, Devesa J, Muller EE (1988). Inhibitory effect of cabergoline on the development of estrogen-induced prolactin-secreting adenomas of the pituitary. Eur J Pharmacol.

[22] Abdel-Salam OME, El-Shamarka ME, Salem NA, Gaafar AEM (2012). Effects of *Cannabis sativa* extract on haloperidol-induced catalepsy and oxidative stress in the mice. EXCLI J.

[23] Salman TM, Olayaki LA, Alagbonsi IA, Oyewopo AO (2016). Spermatotoxic effect of galactose and possible mechanism of action. Middle East Fertil Soc J.

[24] Olayaki LA, Alagbonsi IA, Abdulkadir HO, Idowu FO (2017). Low dose of melatonin ameliorates cryptorchidism-induced spermatotoxicity in rats. J Anat Soc India.

[25] Da Porto C, Decorti D, Tubaro F (2012). Fatty acid composition and oxidation stability of hemp (*Cannabis sativa* L.) seed oil extracted by supercritical carbon dioxide. Ind Crop Prod.

[26] Deferne JL, Pate DW (1996). Hemp seed oil: a source of valuable essential fatty acids. J Int Hemp Assoc.

[27] Rausch P (1995). Verwendung von hanfsameno in der kosmetik. Bioresource hemp.

[28] Westerman D, Santos R, Bosley J, Rogers J, Al-Duri B (2006). Extraction of Amaranth seed oil by supercritical carbon dioxide. J Supercrit Fluids.

[29] Rubio-Rodríguez Nuria, de Diego Sara M., Beltrán Sagrario, Jaime Isabel, Sanz Maria Teresa, Rovira Jordi (2008). Supercritical fluid extraction of the omega-3 rich oil contained in hake (Merluccius capensis–Merluccius paradoxus) by-products: Study of the influence of process parameters on the extraction yield and oil quality. The Journal of Supercritical Fluids.

[30] Al-Mutairi KA (2017). Influence of soil physical and chemical variables on species composition and richness of plants in the arid region of Tabuk, Saudi Arabia. Ekologia (Bratislava).

[31] Kirk ES, Graham JK, Squires EL (2001). Increasing membrane cholesterol content benefits the motility of cooled equine semen. Anim Reprod Sci.

[32] Schmid-Lausigk Y, Aurich C (2014). Influences of a diet supplemented with oil and antioxidants on quality of equine semen after cooling and cryopreservation during winter. Theriogenology..

[33] Grady ST, Scott BD, Brinsko DW, Forrest DW, Sawyer JE, Cavinder CA (2009). Dietary supplementation of 2 sources of omega-3 fatty acids and subsequent effects on fresh, cooled, and frozen seminal characteristics of stallions. J Equine Vet Sci..

[34] Rodrigues PG, de Moura RS, Rocha LGP, Bottino MP, Nichi M, Maculan R, Bertechini AG, Souza JC (2017). Dietary polyunsaturated fatty acid supplementation improves the quality of stallion cryopreserved semen. J Equine Vet Sci.

[35] Salman TM, Alagbonsi IA, Feyitimi AA (2018). Role of reactive oxygen species-total anti-oxidant capacity status in *Telfairia occidentalis* leaves*-*associated spermatoprotective effect: a pointer to fatty acids benefit. J Basic Clin Physiol Pharmacol.

[36] Battista N, Rapino C, Di Tommaso M, Bari M, Pasquariello N, Maccarrone M (2008). Regulation of male fertility by the endocannabinoid system. Mol Cell Endocrinol.

[37] Francavilla F, Battista N, Barbonetti A, Vassallo MR, Rapino C, Antonangelo C, Catanzaro G, Barboni B, Maccarrone M (2009). Characterization of the endocannabinoid system in human spermatozoa and involvement of transient receptor potential vanilloid 1 receptor in their fertilizing ability. Endocrinology.

[38] Agirregoitia E, Carracedo A, Subiran N, Valdivia A, Agirregoitia N, Peralta L, Velasco G, Irazusta J (2010). The CB(2) cannabinoid receptor regulates human sperm cell motility. Fertil Steril.

[39] Ramadan MF, Moersel JT (2006). Screening of the antiradical action of vegetable oils. J Food Comp Anal.

